# Identifying Therapeutic Targets for Amyotrophic Lateral Sclerosis Through Modeling of Multi-Omics Data

**DOI:** 10.3390/ijms26157087

**Published:** 2025-07-23

**Authors:** François Xavier Blaudin de Thé, Cornelius J. H. M. Klemann, Ward De Witte, Joanna Widomska, Philippe Delagrange, Clotilde Mannoury La Cour, Mélanie Fouesnard, Sahar Elouej, Keith Mayl, Nicolas Lévy, Johannes Krupp, Ross Jeggo, Philippe Moingeon, Geert Poelmans

**Affiliations:** 1 Servier Research & Development, 91190 Saclay, France; francoisxavier.blaudindethe@servier.com (F.X.B.d.T.); philippe.moingeon@universite-paris-saclay.fr (P.M.); 2 Donders Institute for Brain, Cognition and Behaviour, Radboud University, 6525 EN Nijmegen, The Netherlands; koen.klemann@radboudumc.nl (C.J.H.M.K.);; 3 Department of Medical Neuroscience, Radboud University Medical Center, 6525 EN Nijmegen, The Netherlands; 4 Servier Data Factory, 92284 Suresnes, France

**Keywords:** amyotrophic lateral sclerosis (ALS), multi-omics modeling, molecular landscape, patrimony, therapeutic targets, MATR3

## Abstract

Amyotrophic lateral sclerosis (ALS) is a progressive neurodegenerative disease that primarily affects motor neurons, leading to loss of muscle control, and, ultimately, respiratory failure and death. Despite some advances in recent years, the underlying genetic and molecular mechanisms of ALS remain largely elusive. In this respect, a better understanding of these mechanisms is needed to identify new and biologically relevant therapeutic targets that could be developed into treatments that are truly disease-modifying, in that they address the underlying causes rather than the symptoms of ALS. In this study, we used two approaches to model multi-omics data in order to map and elucidate the genetic and molecular mechanisms involved in ALS, i.e., the molecular landscape building approach and the Patrimony platform. These two methods are complementary because they rely upon different omics data sets, analytic methods, and scoring systems to identify and rank therapeutic target candidates. The orthogonal combination of the two modeling approaches led to significant convergences, as well as some complementarity, both for validating existing therapeutic targets and identifying novel targets. As for validating existing targets, we found that, out of 217 different targets that have been or are being investigated for drug development, 10 have high scores in both the landscape and Patrimony models, suggesting that they are highly relevant for ALS. Moreover, through both models, we identified or corroborated novel putative drug targets for ALS. A notable example of such a target is MATR3, a protein that has strong genetic, molecular, and functional links with ALS pathology. In conclusion, by using two distinct and highly complementary disease modeling approaches, this study enhances our understanding of ALS pathogenesis and provides a framework for prioritizing new therapeutic targets. Moreover, our findings underscore the potential of leveraging multi-omics analyses to improve target discovery and accelerate the development of effective treatments for ALS, and potentially other related complex human diseases.

## 1. Introduction

Amyotrophic lateral sclerosis (ALS), also referred to as Lou Gehrig’s disease, is a severe and progressive neurodegenerative disorder that mainly affects motor neurons, causing them to slowly deteriorate and die [1]. As motor neurons transmit signals from the brain to the musculature to enable (in)voluntary movement, patients gradually lose muscle control as the disease progresses, leading to progressive paralysis, as well as difficulties with speaking, swallowing, and eventually breathing. The disease usually results in a much-shortened life expectancy and premature death, with most people dying from respiratory failure within 2 to 5 years of diagnosis [2].

Although some progress has been made in recent years, the underlying genetic and molecular mechanisms of ALS remain largely unknown. Approximately 10% of ALS cases are familial, and about 70% of familial forms of ALS are caused by rare inherited mutations in a relatively small number of genes. However, the majority of ALS cases are sporadic, i.e., together with environmental and lifestyle risk factors, the onset of disease is associated with a large number of common genetic variants, each with a slightly increased disease risk [2,3]. The pathophysiology of ALS involves the accumulation of abnormal protein aggregates, leading to motor neuron dysfunction and, eventually, neuronal death. These aggregates include misfolded forms of the TAR DNA-binding protein 43 (TDP-43), superoxide dismutase 1 (SOD1), and fused in sarcoma (FUS) proteins, among others [1]. Currently, the few available ALS treatments help in managing symptoms but have a very limited impact on disease progression, and therefore offer only limited and temporary relief to patients. Recently, the first disease-modifying treatment was approved for a rare familial form of ALS due to mutations in SOD1 [4]. Nevertheless, in light of the significant unmet medical need, finding new targets for ALS is critical to develop more effective treatments. Most particularly, developing targeted therapies that address the underlying causes of ALS, such as reducing protein aggregation or protecting (motor) neurons, could dramatically improve the prognosis and quality of life of individuals affected by this disease [5,6]. Therefore, a better understanding of the genetic and molecular mechanisms contributing to ALS is necessary, with the aim of identifying new and highly relevant therapeutic targets. In this respect, modeling approaches can help us to establish a comprehensive understanding and representation of ALS pathogenicity by integrating and analyzing large and diverse sets of multi-omics and (pre)clinical data. As such, these modeling approaches—that incorporate artificial intelligence (AI)—can greatly improve drug discovery by helping to select highly relevant therapeutic target(s), a key step to reduce failure rates during subsequent drug development in patients.

In this study, we used two complementary modeling strategies (the DTID molecular landscape building method and the Patrimony computational platform based on a knowledge graph, see Section 4), based on distinct sets of omics input data and analytical methods, as well as different criteria for prioritizing relevant target candidates. In this hypothesis-free, multipronged approach to identify and represent the perturbed biological processes in ALS, we illustrate how two orthogonal approaches to model disease complexity can provide convergent, as well as distinct, hypotheses regarding novel and relevant therapeutic targets, hence increasing the chances of finding better drug candidates for this devastating disease.

## 2. Results

### 2.1. Candidate Genes/Proteins in the Molecular Landscape of ALS

**Figure 1:**
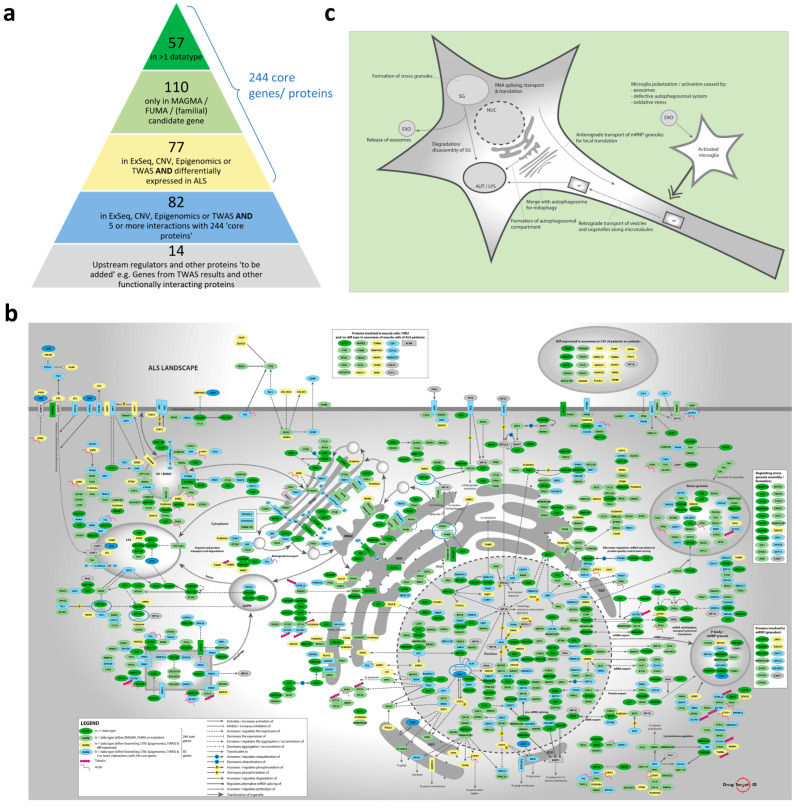
Description of the ALS landscape 2.0. (**a**) Composition of the list of 326 ‘color-coded’ candidate genes/proteins—dark green, light green, yellow, and blue—plus an additional 14 ‘grey’ candidate genes that were prioritized for building the ALS landscape (see also Section 4). (**b**) Molecular landscape of ALS in a motor neuron. (**c**) Schematic representation of the molecular landscape of ALS, with its main functional themes. High-quality/-resolution versions of (**a**–**c**) are also provided as Figures S1, S3, and S5.

The omics data that were collected and used/analyzed to build the molecular landscape of ALS are shown and described in Section 4 and Table S1. Combining the associated genes from the MAGMA and FUMA analyses with the significantly associated genes from other genomic and epigenomic studies resulted in a list of 753 unique protein-coding input genes for the molecular landscape (Table S2). Many of these genes had multiple types of evidence linking them to ALS through different types of omics data, such as GWAS, CNV, and epigenomic data. This large number of input genes was narrowed down. First, all genes associated with ALS through two or more omics data types were included (57 genes), followed by all other genes associated through the MAGMA and/or FUMA analyses (110 genes). Genes that were only found through/in exome sequencing studies, CNVs, epigenomic studies, or TWASs were selected if they were also differentially expressed in ALS (using genome-wide expression data, 77 genes). This led to a ‘core’ gene list of 244 genes. Additional genes/proteins from the list of 753 were included if they had at least five experimental evidence-based interactions (such as binding, expression regulation, activation, inhibition, phosphorylation, translocation, secretion, etc.) with the 244 core genes/proteins (82 genes). This resulted in a list of 326 unique candidate genes/proteins for the molecular landscape (Table S3). Lastly, another 14 genes were added to the list of genes, which added up to a total of 340 candidate genes/proteins for the molecular landscape (Table S3). These 14 additional genes were ‘upstream regulators’ identified by analysis of the 326 genes/proteins (see below), as well as other proteins that were unrelated to ALS but strongly interacted with the 326 genes/proteins. The collection of all 340 candidate genes for the landscape and the data through which they are associated with ALS is visualized and color-coded in the ‘pyramid’ shown in Figure 1a (of which a high-quality/-resolution version is also provided as Figure S1).

### 2.2. Tissue Specificity Analyses

As described in Section 4, we also used FUMA to conduct tissue specificity analyses using ALS GWAS summary statistics. The tissue distribution was consistent across all analyzed ALS GWASs (i.e., the same tissues appeared on top in the different analyses) and revealed that expression in especially the cerebellum, cerebellar hemisphere and brain cortex is linked to ALS GWAS genes. However, as shown in Figure S2, only in the largest and most heterogeneous GWASs (by Van Rheenen et al., 2021 (without the Asian cases) [7] and Nicolas et al., 2018 [8]) did the tissue specificity analyses of these tissues meet statistical significance. This is probably due to the smaller sample size of the other GWASs and/or ethnic heterogeneity.

### 2.3. Upstream Regulator Analyses

Upstream regulator analyses were performed on the list of 753 input genes and the smaller list of 326 candidate genes (see Section 4).

The female sex hormone beta-estradiol was the main upstream regulator in both analyses, but it was much more significant in the smaller list of candidate genes (*p* = 8.60 × 10^−11^ compared to *p* = 2.37 × 10^−4^) (Table S4).

### 2.4. Molecular Landscape and Identification/Selection of Therapeutic Targets

All possible interactions (e.g., binding, (de)phosphorylating, activating, inhibiting, expression regulation, (de)ubiquitinating, (de)methylating, degrading, transporting, etc.) among the 340 candidate proteins for the landscape were compiled through systematic analysis of the literature and PPI databases. Only genes/proteins with at least one interaction with another landscape gene/protein were included in the landscape, with all interactions being based on experimental literature evidence and not on (in silico) predictions, which resulted in 293 landscape genes/proteins (Table S5).

The 293 interacting proteins in the molecular landscape of ALS regulate multiple signaling cascades/pathways and processes. In Figure 1b—of which a high-quality/-resolution version is also provided as Figure S3—the landscape proteins and interactions in a motor neuron are shown, while the landscape proteins that mainly interact in microglial cells are shown in Figure S4. Within the landscape, we identified six main functional themes, each containing several interacting signaling cascades/pathways:

1. Cytoskeleton-dependent transport of organelles and mRNP/stress granules.
2. ER-ERGIC-Golgi regulation and function, including vesicle transport from and to the Golgi apparatus, Golgi acidification, and (indirect) involvement in Golgi fragmentation.
3. Autophagosomal function, which includes autophagy, mitophagy, autophagosome-lysosome fusion, lysosomal function, and ‘autophagic flux’ in general.
4. Hypoxia, which involves oxidative stress and processes that are protective in cerebral ischemia and ischemic stroke.
5. mRNP/stress granules, which includes the expression or functional involvement in the regulation of mRNP and/or stress granules.
6. Microglial regulation, which involves the polarization, activation/inhibition, pyroptosis, and (programmed) death of microglia.

In Figure 1c—of which a high-quality/-resolution version is also provided as Figure S5—a schematic representation of the landscape, with its main functional themes, is shown. As further detailed in Section 4, after building the molecular landscape of ALS, we scored all the landscape proteins based on several criteria, and we identified 47 ‘top’ targets for further follow-up, i.e., targets with a score ≥5 (Table S6). Targets that were too generic (such as STAT3) or had already been strongly linked to ALS (through previous extensive research) and therefore no longer qualified as ‘novel’ therapeutic targets (such as TDP-43, SOD1, FUS, C9ORF72, VCP, etc.) were removed from this list in advance.

### 2.5. Cross Analysis of the ALS Landscape with Patrimony

To take advantage of the different strengths of the two modeling approaches and their unique features, we further evaluated the output of the ALS landscape with the output from the Patrimony method. The first step was to compare the score of each gene/protein in the ALS landscape with its main score from Patrimony ALS, i.e., the score related to biological relevance. This score is the average of all the scores in Patrimony (genetic, transcriptomic, and aggregation) for each gene and its network (see Section 4). In this respect, and in Table S7, we have provided the landscape and Patrimony scores for all 293 landscape proteins (Table S5) and the 47 ‘top’ (putative) targets from the landscape for further follow-up (Table S6).

**Figure 2:**
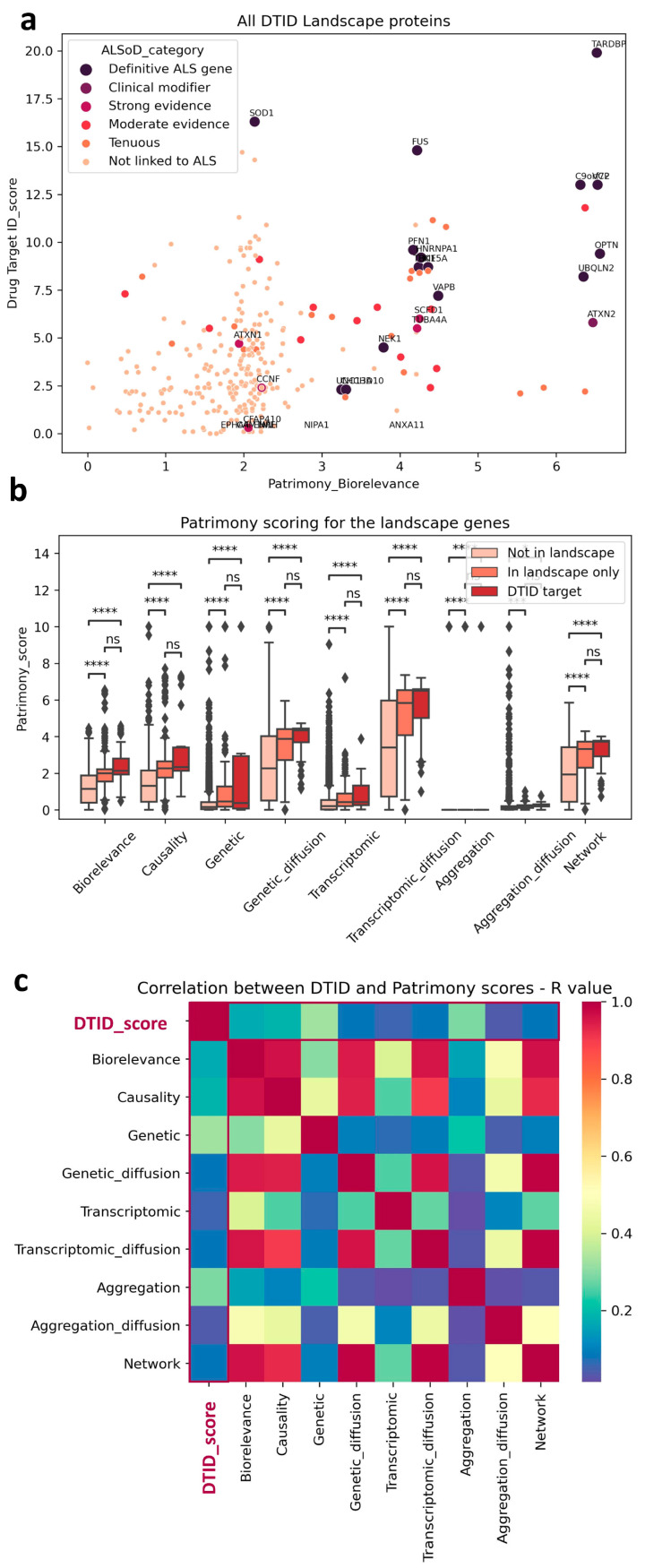
Scoring comparison between DTID and Patrimony. (**a**) Trend correlation between DTID landscape and Patrimony biorelevance scores, highlighting genes with a known genetic link with ALS. (**b**) Genes included in the DTID ALS landscape have significantly higher scores in Patrimony. Despite not being significant, a tendency to a higher score can be observed for the 47 potential targets identified in the DTID landscape; *** *p* < 0.001, **** *p* < 0.0001. (**c**) Correlation matrix between the DTID and Patrimony scores.

We decided to use generic ALS, i.e., all ALS forms, sporadic or familial with TDP-43 pathology (excluding SOD1 and FUS familial forms that have little or no TDP-43 aggregation [9]). Interestingly, we observed a trend toward correlation (correlation coefficient = 0.169611; *p*-value = 6.139117 × 10^−132^) between the two scores when they were plotted against each other (Figure 2a), with some notable examples.

For example, TARDBP (TPD-43) has the highest score in both models, which corroborates its known central role in disease pathophysiology [10]. In contrast, SOD1 has a much lower score in the Patrimony model, because it is associated with a disease form lacking TDP-43 pathology, a distinction not accounted for in the ALS landscape. However, as expected, its score is very high for SOD1 ALS in Patrimony. In general, genes that have a strong genetic connection to ALS were ranked highly by both tools (See Figure 2a). Genes/proteins in the ALS landscape also have a higher score in Patrimony for all categories (Figure 2b) and this is even stronger, albeit not significant, for the 47 ’top’ landscape targets (see above; named ‘DTID targets’ in Figure 2b). The difference between these 47 targets and the rest of the landscape is smaller for the genetic score, due to the selection criteria for novel targets that removed the most genetic (and well known) targets such as TDP-43, SOD1, FUS, C9ORF72, VCP, etc. from this list (see above). Nevertheless, this indicates that the landscape allowed us to prioritize more novel genetically-linked targets than just the familial ALS genes. Importantly, both methods are independent, as the landscape scores are only weakly correlated with any Patrimony score, as measured by R value in a Pearson correlation (Figure 2c).

### 2.6. Relevance of Targets Already Explored by the Pharmaceutical Industry

**Figure 3:**
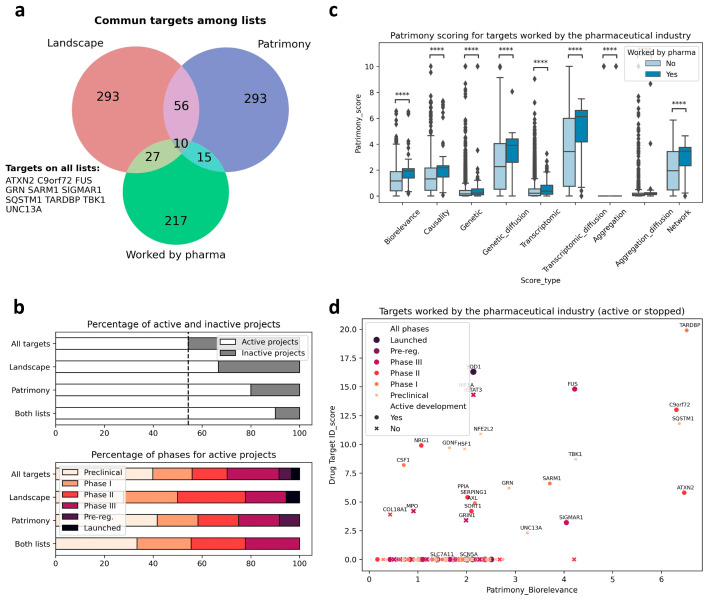
Targets worked on by the pharma industry in ALS in the landscape and Patrimony models. (**a**) Overlap among the targets worked on by the pharmaceutical industry, the ALS landscape genes, and the corresponding top 293 targets for the Patrimony biorelevance score. The names of the 10 targets common to all lists are displayed. (**b**) Top panel: genes on the Patrimony list or the DTID list have a higher proportion of targets for which drug development is ongoing. The proportion is even higher for targets on both lists. Bottom panel: no obvious difference in pharmaceutical phases can be seen between the different target lists. (**c**) Targets worked on by the pharma industry have significantly higher scores in Patrimony; **** *p* < 0.0001. (**d**) Active and inactive targets worked on by the pharma industry have higher scores in both the Drug target ID landscape and Patrimony platform. The highest pharmaceutical phase attained is represented by a color code.

**Figure 4:**
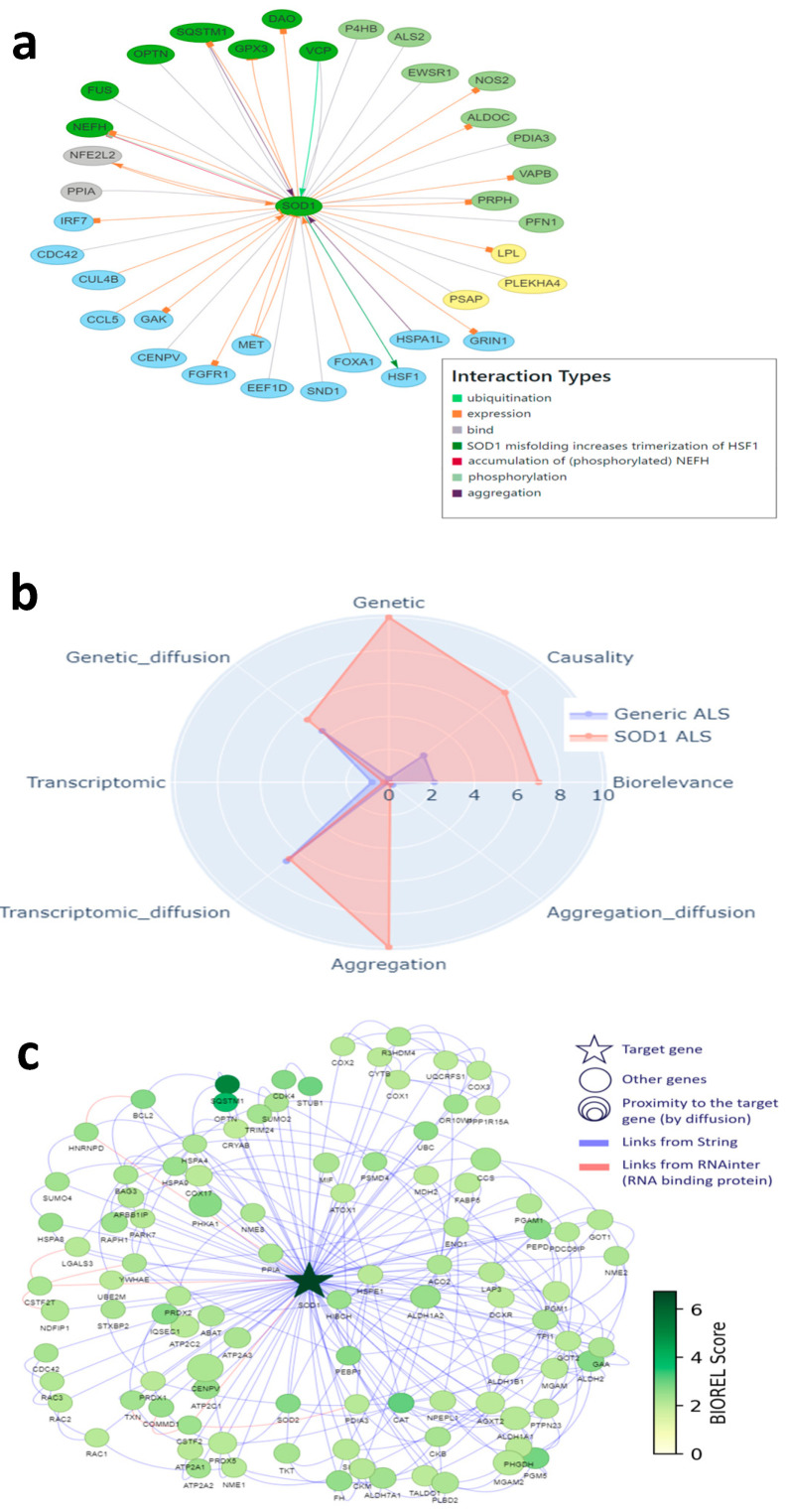
SOD1, a well-known target, in the DTID landscape and Patrimony. (**a**) Interactions of SOD1 with other proteins in the DTID landscape; the color code corresponds to that in Figure 2a. (**b**) SOD1 scoring in Patrimony for generic or SOD1-related ALS. (**c**) Protein–protein interaction network of SOD1 constructed using the Patrimony list, with the corresponding biorelevance scores, for SOD1-related ALS.

We then used the two modeling approaches to evaluate the relevance of targets explored by the pharmaceutical industry to develop new treatments against ALS (i.e., target validation), and also to identify new targets. We identified 217 different targets from the ALS competitive environment that have been, or are being, considered for drug development, between 1989 and March 2025 (https://clinicalintelligence.citeline.com/drugs/, accessed on 5 March 2025). Of the 217 industry targets identified, 27 are present in the landscape, which itself comprises a total of 293 proteins. For Patrimony, we compared the 27 genes encoding these targets to the 293 genes with the highest biological relevance score for generic ALS, and 15 of the 217 industry targets are also on this list, including 5 that are absent from the landscape, showing the importance of using complementary disease models. Nevertheless, 10 targets explored by the pharmaceutical industry are common between the landscape and Patrimony, suggesting their central relevance to ALS (Figure 3a). Genes present in both the landscape and Patrimony lists, and to a lesser extent genes that are present in only one list, are enriched for targets that are still in active drug development (Figure 3b, top panel), showing the relevance of using two modelling approaches. Such a clear difference is not as evident when considering the development phases of the targets in such lists (Figure 3b, bottom panel). Similarly, ALS drug targets have higher scores for all measurements in Patrimony (Figure 3c). When assessing the scores of these industry targets in both models, two groups can be distinguished: targets with a high score in either Patrimony or the landscape, and targets with a low score in both models (Figure 3d). Among the latter category of genes, neither *SCN5A* nor *SLC7A11*, which encode proteins targeted by Riluzole, a drug approved for ALS treatment [11], are found in the landscape, as they are not genetically linked to ALS and encode proteins modulated as part of a symptomatic treatment for ALS [11,12]. On the other hand, many targets with effective drugs in advanced clinical stages score well on either tool, again demonstrating the value of using multiple disease models (Figure 3d). A notable example is SOD1, for which the antisense oligonucleotide drug Tofersen was recently approved for use in patients with ALS related to SOD1 [4]. In the landscape, the important role of SOD1 is reflected by its ‘interaction network’, i.e., the many different functional interactions it has with other landscape proteins (Figure 4a). In Patrimony, the genetic and aggregation scores for SOD1 are high for SOD1 ALS and low for generic ALS, indicating that different ALS forms need to be modeled (Figure 4b). Additionally, some other scores, such as genetic diffusion and transcriptomic diffusion, are also high in both forms (Figure 4b), which shows that SOD1 interacts with several targets that either have genetic links to the disease or exhibit differential expression in ALS (Figure 4c), allowing comprehensive assessment of SOD1′s role in ALS pathology in patients with specific genetic mutations.

### 2.7. Corroboration of MATR3 as a New Target of Interest

**Figure 5:**
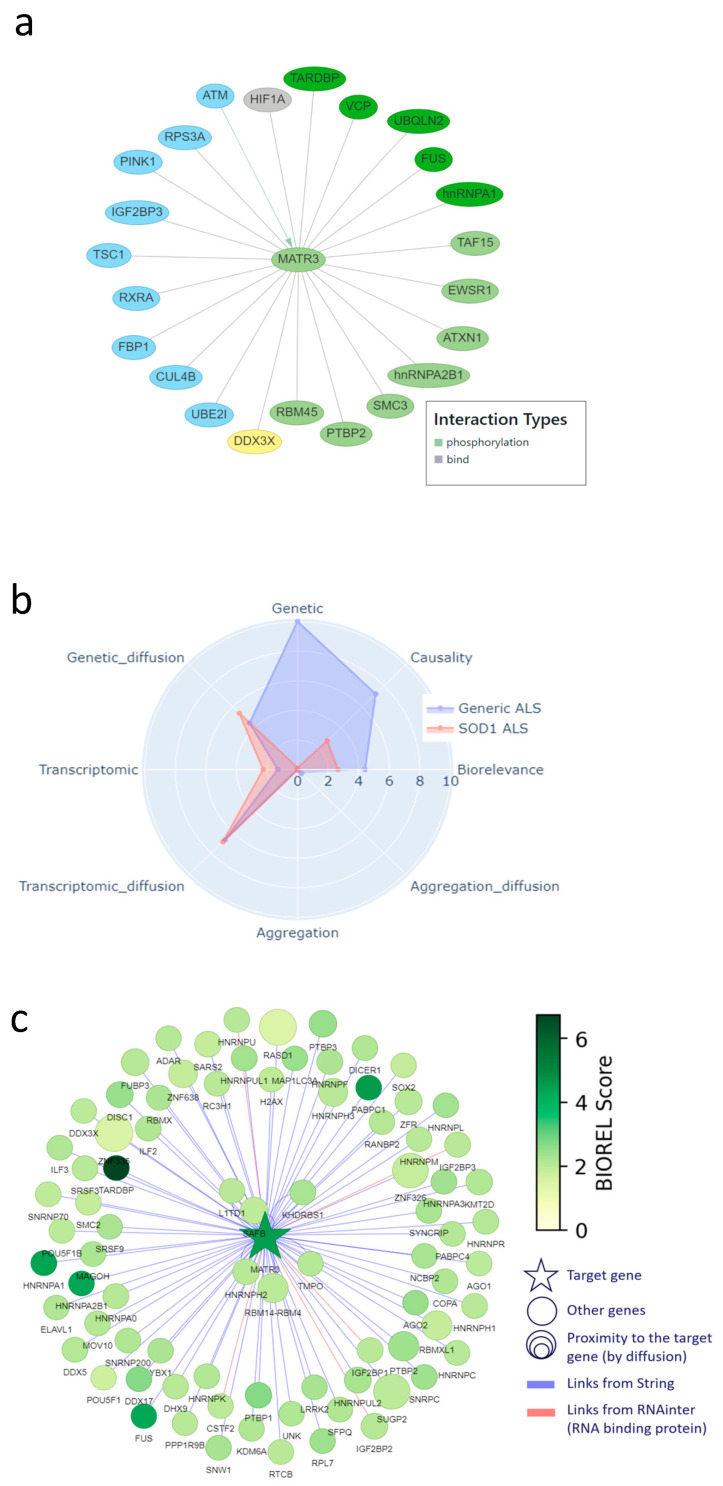
MATR3, corroborated as a novel target in the DTID landscape and Patrimony. (**a**) Interactions of MATR3 with other proteins in the DTID landscape; the color code corresponds to that in Figure 2a. (**b**) MATR3 scoring in Patrimony for generic or SOD1-related ALS. (**c**) Protein–protein interaction network of MATR3 constructed using the Patrimony list, with the corresponding biorelevance scores, in generic ALS.

To illustrate how the two-pronged modeling approach described above can also identify or corroborate novel therapeutic targets for ALS, and to show the level of information provided, we chose MATR3 (Matrin-3) as an example. MATR3 was among the highest scoring targets in both models. MATR3 is a nuclear RNA- and DNA-binding protein with roles in transcription, nuclear retention of defective RNAs, and regulation of innate immunity [13]. In addition, MATR3 has multiple biological/functional interactions with other ALS-linked landscape proteins, including key ALS genes such as TARDBP, FUS, VCP, HNRNPA1, and HNRNPA2B1 (Figure 5a). Moreover, MATR3 has been linked to both familial [14] and sporadic ALS [15].

In addition, we found experimental evidence for all five aspects of target specificity for MATR3 in ALS. First, MATR3 has regional specificity: its expression changes in the motor cortex of sporadic, spinal ALS patients compared to controls [16], and it is also differentially expressed in exosomes derived from the muscle cells of these patients [17]. Moreover, MATR3 deficiency leads to loss of motor neurons and cerebellar Purkinje cells [18,19,20]. Second, MATR3 shows temporal specificity, i.e., its loss of function is related to early-stage ALS features [18]. Third, MATR3 has symptomatic specificity, because it is linked to spinal ALS [21]. Fourth, MATR3 shows molecular specificity, because, as shown in Figure 1b and Figure 5a, it interacts with multiple other landscape proteins and, as such, is involved in several landscape themes. Finally, MATR3 shows modulatory specificity, as a recent study indicates that (early) reduction or removal of MATR3 in/from the nucleus could decrease ALS pathology and symptoms [22].

In addition, the Patrimony model confirms that MATR3 is important for general ALS, but not so much for SOD1 ALS, even though it has slightly higher scores for transcriptomic and genetic network scores, reflecting its vicinity to several genes that are prominent in the disease (Figure 5b). The graph of protein–protein interactions included in Patrimony further illustrates that MATR3 interacts with key ALS genes and shows clear overlap with the interaction network in Figure 5a, but also shows that both methods identify a distinct set of interacting proteins (Figure 5c), which again shows the complementarity of the two models.

### 2.8. Combining the Two Modeling Approaches

**Figure 6:**
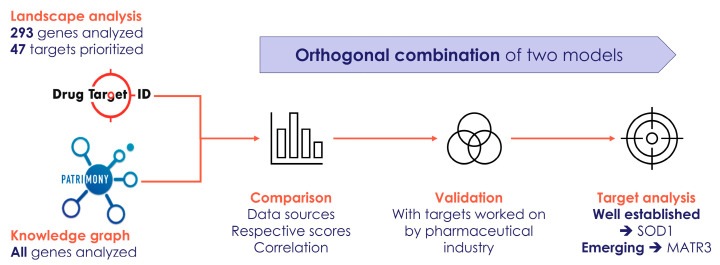
Flowchart that summarizes how we have combined two modeling approaches to identify and corroborate novel therapeutic targets for ALS.

Lastly, in Figure 6, we have provided a flowchart that summarizes how we have combined the two modeling approaches (the DTID molecular landscape building method and the Patrimony computational platform) to identify and corroborate novel therapeutic targets for ALS.

## 3. Discussion

Modeling approaches like the ones described and applied in this study are powerful tools to help discover pertinent new therapeutic targets, by generating a comprehensive hypothesis-free representation of ALS as a perturbed biological system. By examining these models, inferences can be made regarding the key drivers of the disease, identifying nodes and pathways that are most disrupted and—when they have good modulatory and molecular specificity—thus represent promising therapeutic targets.

With the aim of identifying relevant ALS targets, we used two modeling approaches to integrate vast amounts of omics data to map the complex interactions and pathways involved in ALS. These two methods were complementary because they rely upon different data sets, analytic methods, and scoring systems to rank target candidates. For example, the landscape approach started from genes/proteins that are disease-specific—i.e., they have already been linked to ALS—and resulted in a biological construct: a large number of interacting proteins in motor neurons and/or microglial cells. In addition, the ALS landscape not only contains ‘basic’ protein–protein interactions (PPIs) where both proteins physically bind each other, but also a large array of other possible PPIs, such as activation, inhibition, expression regulation, phosphorylation, ubiquitination, methylation, transport, etc., which very much adds to the biological and functional validity, as well as the degree to which landscape proteins could be further developed as (novel) drug targets, i.e., their ‘developability’. In contrast, the Patrimony model is initially disease-agnostic in that it starts from (only) basic PPIs for all proteins encoded by genes in the genome and only at a later stage adds disease-specific information. Therefore, from the Patrimony scores, no inferences can be made about the biological/functional validity and (putative) developability of potential ALS targets. However, Patrimony includes data specific to ALS subtypes, therefore allowing differential scoring for different ALS forms.

The tissue specificity analyses in the landscape approach show that identifying ALS as a disease of only motor neurons is an over-simplified representation. Enrichment in the brain cortex, which is where the somas of motor neurons are located, follows the classical hypothesis. However, enrichment for the cerebellum and cerebellar hemisphere indicates that a more complex mechanism is involved, wherein the cerebellum modifies motor activity by comparing the input from the motor cortex with proprioceptive information that it receives back from muscles via the dorsal root ganglion [23,24]. Involvement of the cerebellum in ALS has been suggested before [25,26,27,28], but, based on our analyses, may be more significant than previously thought. More specifically, while structural and functional imaging consistently show cerebellar changes, research and postmortem studies remain heavily focused on supratentorial regions, neglecting the cerebellum’s involvement in ALS, despite growing evidence of its degeneration and contribution to core clinical symptoms such as dysarthria, gait impairment, and pseudobulbar affect. This oversight limits a comprehensive understanding of ALS pathology and underestimates the cerebellum’s compensatory role in response to motor system degeneration, implying that cerebellar involvement in ALS should be considered when developing new therapeutic strategies. Currently, data sets with information regarding tissue-specific, and especially cell type-specific, expression in the human brain are increasing, but are still very limited. Inclusion of these data in our models would improve the biological model for the disease, and would therefore also potentially improve drug target development through tissue-specific or even cell type-specific targeting with precision-medicine modalities such as bispecific antibodies [29] or antibody–drug conjugates [30].

Given the distinct features of the two models, some targets were only highly ranked in one of them, such as the SOD1 gene that is involved in a highly specific familial form of the disease. Of note, scores for SOD1 related to genetic and transcriptomic diffusion were nevertheless also high in Patrimony, indicating that SOD1 interacts with several other targets with either genetic links to the disease or different regulation levels in ALS. This interconnectedness of SOD1 is also visualized in the landscape model, in which SOD1 is included based on its association to ALS through both rare (familial) and sporadic (common) genetic variants. Overall, this functional interaction of SOD1 in both familial and sporadic forms of ALS would argue (in this case) against using completely distinct disease models.

As a result of the above, the orthogonal combination of the two methods led to both significant convergences as well as some complementarity in the outputs, both for identifying and corroborating new targets and validating existing ones. For example, and as detailed above, the two-model approach described here allowed us to identify and/or corroborate novel potential therapeutic targets such as MATR3. As for modulating MATR3, and as already indicated above, it seems that reducing or removing MATR3 in/from the nucleus could decrease early ALS pathology and symptoms. In this respect, approaches aimed at reducing nuclear MATR3 (through nucleus-specific antisense oligonucleotides, ASOs) or promoting the translocation of nuclear MATR3 to the cytoplasm (through small molecules) could be developed into a novel ALS treatment, reinforcing the translational potential of our findings. For validating existing targets, we found that, out of 217 different targets that have been or are being investigated for drug development, 10 have high scores in both the landscape and Patrimony models, suggesting that they are highly relevant for ALS. We found this overlap with the targets being/having been worked on by pharma strikingly low, considering that the landscape contains the 293 top genetically linked targets that interact in a biological construct for ALS. This is partially explained by the fact that many treatments worked on by the pharma industry aim to relieve symptoms and are therefore not necessarily acting on the causal roots of the disease. In addition, a limitation of our study may be that our modeling and the assumptions used are incomplete. Moreover, we may miss a part of the disease-regulating mechanisms that are involved in ALS, as the data sources that we used do not necessarily represent all pathophysiological pathways involved in ALS, and more large-scale multi-omics would support better comprehension of the disease. Therefore, in future versions of these models, more (agnostic) omics data could be added to the KG of the Patrimony model. In addition, the landscape building model could include more types of protein/gene interactions, for example epigenetic regulation or regulation by miRNAs, lincRNAs, and mRNAs. This would provide important new insights, especially in relation to the aggregation of proteins and the mRNP granules in ALS. However, as both our modeling approaches (1) are based on hypothesis-free genome-wide patient data, (2) are curated by bioinformaticians and disease experts to reduce noise and increase biological validity, and (3) overlap more between themselves than with the pharma targets, we assume that this discrepancy is largely due to the fact that targets developed by pharma are/were (mostly) based on candidate gene studies, small cohorts, and targeting symptomatic relief instead of the underlying disease-modifying pathways.

Today, none of these pharma targets have resulted in a disease-modifying drug for sporadic ALS. Accordingly, we believe it is very significant that we have identified—through two complementary models—multiple potential novel drug targets to treat ALS.

## 4. Materials and Methods

### 4.1. Molecular Landscape for the Identification and Selection of Therapeutic Targets

**Figure 7:**
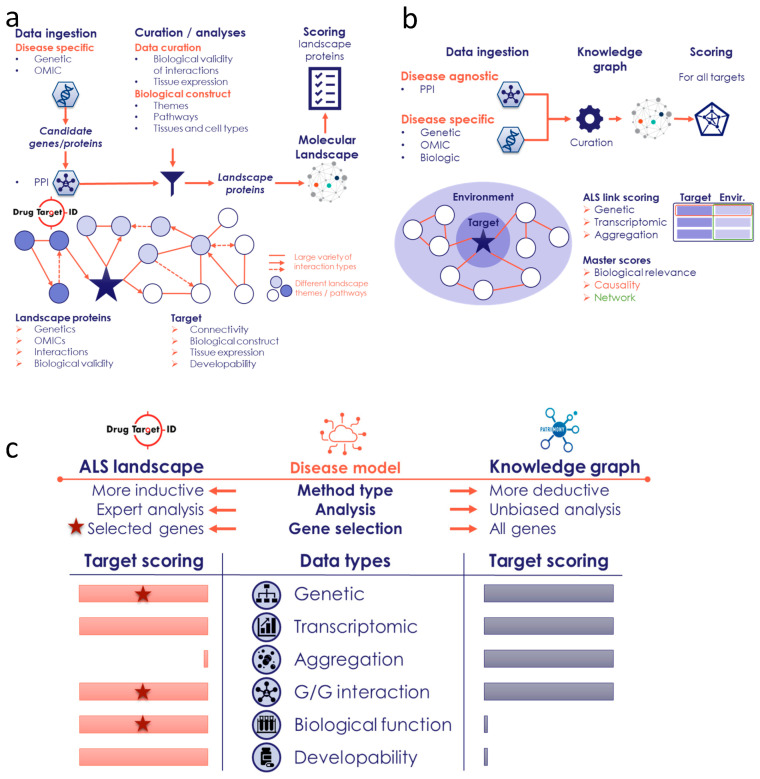
Workflow of the DTID and Patrimony ALS methods. (**a**) Different steps/phases in building the molecular landscape of ALS by DTID, as further described in Section 4. (**b**) The Patrimony knowledge graph, built using both disease-specific and disease-agnostic data, allows for the calculation of ALS relevance scores (genetic, transcriptomic, and aggregation) for all protein-coding genes. Using the protein–protein interaction graphs, the equivalent gene environment scores are calculated as well. (**c**) High-level comparison between the two methods, which differ based on the data used, the analysis, and the number of genes for which a score is calculated. The stars indicate that these data types are used to select landscapes genes.

The molecular landscape building method of Drug Target ID (DTID), Ltd. was developed to integrate several types of genome-wide human omics data with functional data for any complex disease. Thus far, this approach has been applied to several neurological and non-neurological diseases, e.g., Parkinson’s disease [31], Tourette’s disorder [32], pelvic organ prolapse [33], and, most recently, the overlap between Alzheimer’s disease and somatic insulin-related diseases [34]. By using genome-wide omics patient-derived data—such as genomic, epigenomic, transcriptomic, proteomic, and metabolomic data—a built landscape is based on data that are hypothesis-free, i.e., without preconceived biases such as candidate gene studies or other hypothesis-driven research data (Figure 7a). In addition, the omics data that are used for building molecular landscapes are meticulously curated and selected for their robust study designs. This results in a (prioritized) list of candidate genes for the disease of interest through multiple omics data types. The list of candidate genes is subsequently analyzed, using tissue- and cell type-specificity analyses, upstream regulator analyses, and mapping of protein–protein interactions (PPIs), as well as protein–RNA and protein–DNA interactions (Figure 7a). Through the assumption that disease pathology-contributing genes/proteins interact together, the list of candidate genes/proteins can be reduced to a more stringent list of (putative) landscape genes/proteins. At the core of the molecular landscape building method lies the integration of these landscape genes/proteins into a biological construct. This means that all interactions are curated for their biological validity and placed in disease-relevant tissues and cell types.

#### 4.1.1. Collecting/Compiling Omics Input Data for the ALS Landscape

To build the molecular landscape of ALS, all publicly available omics data—including genome-wide association study (GWAS) data, exome sequencing and copy number variation (CNV) data, epigenomic data, transcriptome-wide association study (TWAS) data, and transcriptomic and proteomic data—were collected to compile a list of ALS-associated candidate genes for the landscape. These omics input data for the ALS landscape were collected through a systematic search using PubMed (https://pubmed.ncbi.nlm.nih.gov/, accessed on 8 January 2025). For each data type, a specific search query was used, and the search results were screened for inclusion/exclusion as input data. The papers from the search results were screened and excluded/included based firstly on their title and abstract, secondly on the (availability of the) full text in English, and thirdly on the accessibility, the overlap of the (summary) data, and the presence of genome-wide significant hits. In Table S1, the included studies per omics data type are shown, as well as the number of genes that were used as input genes for the landscape.

#### 4.1.2. MAGMA and FUMA Analyses of GWAS Data

Using the available summary statistics of the GWAS data (see Table S1), we then performed MAGMA analyses—for all SNPs within each gene—and FUMA analyses to obtain lists of associated genes.

First, we used Multi-marker Analysis of GenoMic Annotation (MAGMA) [35] to perform gene-based analysis of the summary statistics of the GWAS data. MAGMA combines all variants that are mapped to a gene, while adjusting for the linkage disequilibrium (LD) between those variants, and tests the joint association of all variants in the gene with the phenotype, resulting in a single, gene-wide *p*-value for each gene. This approach reduces the number of tests that need to be performed and enables the identification of effects consisting of multiple weaker associations that would be missed in the individual variant analysis. Specifically, for each of the 19,427 protein-coding genes included in the NCBI 37.3 database, we considered all single nucleotide polymorphisms (SNPs) located within each gene. Then, using an updated SNP-wise Mean model, we combined the resulting SNP *p*-values into a gene test statistic (the sum over squared SNP Z-statistics) and computed the corresponding gene-wide *p*-value. We used the 1000 Genome Project Phase 3 European population as reference data to account for the LD-induced covariance of SNP *p*-values. All genes from the MAGMA analyses with a significant gene-wide *p*-value—i.e., FDR *p* < 0.05 (which corresponds to a *p* = 0.05/19427 = 2.57E-06)–were considered as input genes for the landscape, which resulted in 38 unique genes.

We also used FUMA [36] to map functionally annotated SNPs to genes by combining three mapping strategies: positional, eQTL (expression quantitative trait locus, i.e., a SNP that is not located inside a gene but influences the expression of this gene ‘from a distance’), and chromatin interaction mapping. These three types of functionally annotated SNPs are also referred to as xQTLs (x quantitative trait loci), and we limited the FUMA analyses to brain xQTLs. Genes that met the significance criteria for each of the three xQTL types (e.g., FDR *p* < 0.05 for eQTLs) were considered input genes for the landscape, which resulted in 81 unique genes.

#### 4.1.3. Tissue Specificity Analyses of GWAS Data

We also used FUMA [36] to perform tissue specificity analyses in FUMA using the available summary statistics of the GWAS data and standard settings.

#### 4.1.4. From Input Genes to Candidate Genes

A total of 753 unique genes were associated with ALS through genomic/epigenomic data (Table S2). Multiple genes were linked to ALS through different types of data, e.g., through GWAS, CNV, and epigenomic data. This list of input genes was reduced to a more stringent list of candidate genes. The candidate genes were selected from the total list of input genes, as follows:

(1) All genes associated through more than one type of genomics data were included (color code: dark green)
(2) Genes associated only through MAGMA or FUMA analyses or identified through a (familial) mutation or mutations were included (color code: light green)
(3) Genes associated through exome sequencing, CNVs, epigenomics, or TWAS studies were included, but only if they were also differentially expressed in ALS motor cortex, spinal cord, or CSF (color code: yellow). Of note, we would also have liked to include differential expression data from the cerebellum of ALS patients vs. controls, but these genome-wide data were not available
(4) Remaining genes associated through exome sequencing, CNVs, epigenomics, or TWAS studies but that were not differentially expressed in ALS were only included if their encoded proteins had at least five interactions with the proteins/mRNAs encoded by the 244 genes from points 1–3 (color code: blue)
(5) Lastly, genes/proteins not genetically associated with ALS were added. These include the two most significantly enriched upstream regulators (from the IPA analysis, see below)—i.e., GRN and LDB1—and genes coding for proteins that were strongly regulated/interacting in the landscape (color code: grey genes)
(6) This prioritization process resulted in a list of 340 candidate genes for the ALS landscape, i.e., 326 candidate genes with a genetic link to ALS, and 14 ‘grey’ candidate genes without a known link to ALS (Table S3)

#### 4.1.5. Upstream Regulator Analyses

We also used Ingenuity Pathway Analysis (IPA) (QIAGEN, Aarhus, Denmark) (https://digitalinsights.qiagen.com/products-overview/discovery-insights-portfolio/analysis-and-visualization/qiagen-ipa/, accessed on 8 January 2025) to identify upstream regulators–proteins and other molecules (such as drugs and metabolites) that regulate the expression of a significant number of genes/proteins from a given list—that are enriched within the total list of 753 input genes (Table S2) and the smaller list of 326 candidate genes (Table S3). As can be derived from Table S4, the upstream regulators beta-estradiol and progranulin (GRN) are significant in both analyses and show more significant enrichment (a lower *p*-value) in the smaller list of 326 candidate genes.

#### 4.1.6. From Candidate Genes to Landscape Genes/Proteins

We assume that genes/proteins associated with ALS converge on similar signaling cascades, pathways, and themes in the landscape. Therefore, in addition to the above-described analyses, we performed an extensive literature search on the functions and interactions of (the proteins encoded by) the 340 candidate genes (Table S3). Dark green, light green, and yellow genes/proteins were excluded from the candidate list when they have no interactions with other green, yellow, or blue candidate genes/proteins or the two upstream regulators GRN and LDB1 and are not part of a group of proteins with a similar function. In this respect, a total of 47 genes/proteins—11 dark green genes/proteins, 24 light green genes/proteins and 12 yellow genes/proteins—were removed from the list of candidate genes. This led to a final list of 293 landscape genes/proteins (Table S5) that all encode proteins with (a) function(s) and/or interaction(s) in the ALS landscape. Importantly, all possible interactions (e.g., binding, (de)phosphorylating, activating, inhibiting, expression regulation, (de)ubiquitinating, (de)methylating, degrading, transporting, etc.) between the landscape proteins were based on experimental literature evidence, and not on, e.g., in silico predictions of these interactions taking place or predicted co-expression.

#### 4.1.7. Molecular Landscape of ALS

The interactions among the 293 landscape genes/proteins were visualized in figures in a motor neuron and a microglial cell, the two main cell types implicated in ALS. Some of the interactions are not shown in these figures (1) because the proteins that interact are both subunits of the same complex and are therefore shown as such or (2) to limit the number of protein interactions of minor importance (e.g., interactions by proteins that are very generic or have only limited involvement in the landscape processes).

Of note, for all protein–protein interactions, we manually assessed if these were likely/possible to occur. For example, if a pull-down assay (of homogenized cells) showed an interaction between protein A and protein B, we checked if these proteins were located in the same cell compartment (e.g., cytoplasm, or plasma membrane/cytoplasm), and that a binding interaction could therefore physically take place in vivo.

Further, protein and signaling cascades were placed in the microglial cell landscape if they could be specifically linked to microglia-specific processes. Otherwise, when proteins were cell type-independent—e.g., mitochondrial, cytoskeletal, and Golgi-related—they were placed in the motor neuron landscape.

#### 4.1.8. Scoring of ALS Landscape Proteins

To be able to prioritize the proteins from the landscape for therapeutic target development, the 293 landscape proteins from Table S5 were ranked based on—among other items/criteria—their functional involvement and (differential) expression in different ALS-associated cell types and tissues (motor cortex, upper/lower motor neurons, cerebellum, Purkinje cells), their involvement in the six main functional themes emerging from the landscape, and their connectivity (i.e., number of interactions) in the landscape. In this way, we generated scores for all landscape proteins, and from this list of 293 proteins we identified 47 ‘top’ (putative) targets for further follow-up, i.e., targets with a score ≥5, after removing targets that were too generic (such as STAT3) or that have already been strongly linked to ALS and that therefore do not qualify anymore as ‘novel’ therapeutic targets (such as TDP-43, SOD1, FUS, C9ORF72, VCP, etc.) (Table S6).

#### 4.1.9. Identifying Promising Novel Therapeutic Targets

Furthermore, from the landscape proteins with the highest scores, we selected the most promising novel (putative) therapeutic targets based on five broad aspects of target specificity. In this respect, an ideal therapeutic target for ALS should have ´regional specificity´, in that (1) it should be expressed in the cells and tissues involved in ALS—i.e., upper and lower motor neurons, motor cortex, spinal cord and/or cerebellum (and more specifically cerebellar Purkinje cells)—and (2) deficiency/loss of the target should be associated with impaired motor neuron/Purkinje cell functioning. Furthermore, a valid ALS drug target needs to show ‘temporal specificity’, i.e., changes in its expression or functionality are linked to ALS onset and/or progression. Another important aspect is ‘symptomatic specificity’, meaning that impairment of the target should be associated with specific ALS symptoms/types (e.g., bulbar vs spinal) or with ALS symptom severity and progression. In addition, a relevant ALS drug target should have ‘molecular specificity’, in that, when impaired, it should play a key role in causing the disease, reflected by its involvement in multiple protein interactions and functional themes in the molecular landscape. Finally, an ideal ALS target should have ‘modulatory specificity’, i.e., it should be feasible to selectively and safely modulate the putative drug target to slow down or reduce ALS symptoms. Taken together, the most strongly supported therapeutic targets for ALS should adhere to all five aspects of specificity, while (also) being strongly implicated in either familial and/or sporadic ALS.

### 4.2. Patrimony Platform for Target Discovery

Patrimony is a computational platform that uses a knowledge graph (KG) to combine different databases of (major) interest in drug discovery, such as Drugbank [37] (drug–target), DisGenet [38] (gene–phenotype), and the STRING database [39] (gene–gene). This creates a large KG that connects diseases with genes and drugs (Figure 7b). Disease modeling with Patrimony unites and integrates multiple data sources to build support for potential new treatments for complex and multifactorial diseases, by nominating putative therapeutic targets for specific subtypes of patients [40]. Patrimony computes eight different scores for each human gene based on a specific disease. These scores use transcriptomic data (gene dysregulation in target organ(s)) and genetic data, which include GWASs, mutations associated with inherited forms of the disease, and Mendelian randomization results [41] (Figure 7b). The transcriptomic score only uses patient data, in light of the poor translatability of animal models to human disease. The KG notably includes the interaction between genes via a protein–protein interaction (PPI) network based on the STRING database [39] and an RNA–protein interaction network based on the RNAInter database [42]. Both databases were filtered after calculating a network diffusion score through cross-validation based on retrieval of known disease-associated genes, followed by validation of the approach and results by disease experts. For each new potential therapeutic target (gene), its associated network score measures the relevance, or potential interaction between the gene and the disease, by incorporating biological scores derived from the disease knowledge and the scores of neighboring genes within the PPI (Figure 7b). This feature of Patrimony allows for the ranking of more novel targets based on the premise that the proteins encoded by genes important for a disease tend to cluster in the KG, thus forming ‘disease modules’ [43]. Therefore, targets close to many disease-relevant genes have a higher chance of being important for the disease, even if there is no direct evidence to implicate them in the disease’s pathology.

The Patrimony computational platform is based on a KG that was originally created to model autoimmune diseases. Here, specific modifications were made to the Patrimony platform to adapt its scoring for neurodegenerative diseases and to support platform industrialization [44]. The heterogeneous nature of ALS led us to adapt the calculation of target prioritization scores for specific subsets of patients, such as sporadic, TDP43-related, or SOD1-related ALS patients. A score related to protein aggregation was added to reflect the contribution of toxic protein aggregates to the pathophysiology of neurodegenerative diseases. This added score is a measure of the presence of the target in ALS-related aggregates, based on their reported composition in certain forms of the disease [9,10].

### 4.3. Complementarity Between the Two Modeling Approaches

The DTID landscape and Patrimony are two tools that are fully transparent, as they display the links between a specific target and the disease with references to underlying data sources. However, these tools differ significantly in how they use and analyze omics data, which makes them very complementary (Figure 7c). Specifically, the landscape is more a ‘disease-informed’ model and focuses on key targets related to ALS, including a comprehensive literature review to rank the genes/proteins. In contrast, Patrimony is more a ‘disease-agnostic’ model and evaluates every known gene/protein, relying on genome-wide databases for ranking purposes. Consequently, the landscape has selection criteria based on several biological dimensions (Figure 7a,c), and it allows for the ranking of genes/proteins based on their connection to key ALS biological processes and for the inclusion of a developability component (i.e., the degree to which landscape proteins could be further developed as (novel) drug targets), which are both based on a comprehensive literature review. Another notable difference is that, as opposed to the scores for the DTID landscape proteins, Patrimony scores encompass the presence of the target in the aggregates found in postmortem brain tissues from ALS patients.

## 5. Conclusions

Overall, our two modeling approaches generate convergent hypotheses and enhance the likelihood of identifying novel, biologically ‘robust’ therapeutic targets for ALS. We anticipate that (combining) these and other hypothesis-free modeling strategies that integrate large-scale omics and disease-related data will improve the success rate of drug target development and drive advancements in drug discovery for complex human diseases.

## Data Availability

All of the data generated or analyzed during this study and that do not represent a strategic threat for the companies (Servier and Drug Target ID) have been included in this published article and its Supplementary Material Files. Further inquiries can be directed to the corresponding author.

## Supplementary Materials

The following supporting information can be downloaded at: https://www.mdpi.com/article/10.3390/ijms26157087/s1.

## Abbreviations

AI	artificial intelligence
ALS	amyotrophic lateral sclerosis
CNV	copy number variation
DTID	Drug Target ID
FUS	fused in sarcoma
GWAS	genome-wide association study
KG	knowledge graph
MATR3	matrin-3
PPIs	Protein–protein interactions
SOD1	superoxide dismutase 1
TDP-43	TAR DNA-binding protein 43
TWAS	transcriptome-wide association study
