# Supplementary material for: Identifying Therapeutic Targets for Amyotrophic Lateral Sclerosis Through Modeling of Multi-Omics Data
Source: Int J Mol Sci. 2025 Jul 23;26(15):7087. doi: 10.3390/ijms26157087 (PMC12346086; doi:10.3390/ijms26157087)
Supplement: Supplementary file 1 [file ijms-26-07087-s001.zip › 4 Blaudin de Th_et al. Supplementary Figure 4.pdf]

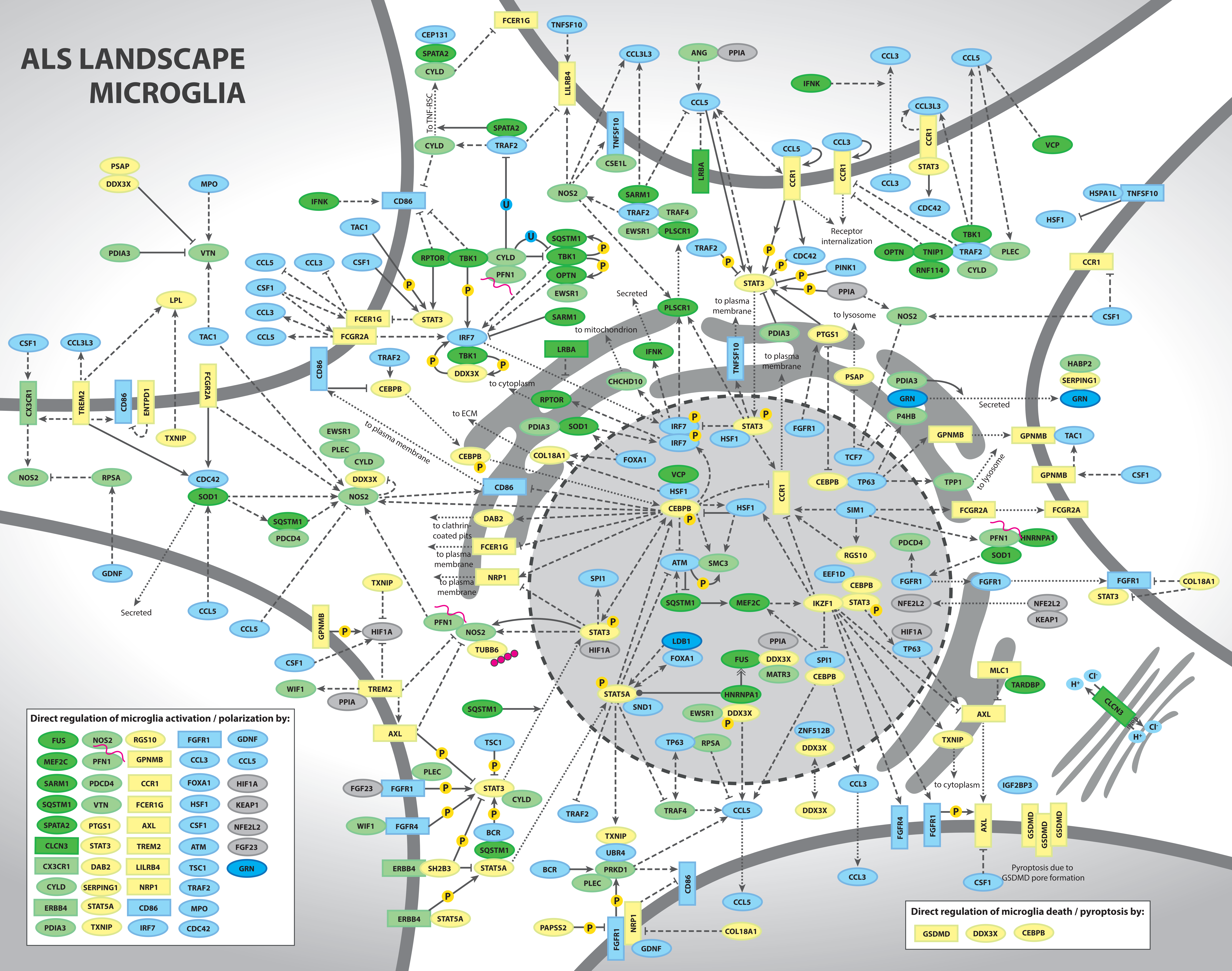
ALS LANDSCAPE
MICROGLIA
Direct regulation of microglia activation / polarization by:
FUS
NOS2
RGS10
FGFR1
GDNF
MEF2C
PFN1
GPNMB
CCL3
CCL5
SARM1
PDCD4
CCR1
FOXA1
HIF1A
SQSTM1
VTN
FCER1G
HSF1
KEAP1
SPATA2
PTGS1
AXL
CSF1
NFE2L2
CLCN3
STAT3
TREM2
ATM
FGF23
CX3CR1
DAB2
LILRB4
TSC1
GRN
CYLD
SERPING1
NRP1
TRAF2
ERBB4
STAT5A
CD86
MPO
PDIA3
TXNIP
IRF7
CDC42
Direct regulation of microglia death / pyroptosis by:
GSDMD
DDX3X
CEBPB
Pyroptosis due to
GSDMD pore formation
To TNF-RSC
Receptor
internalization
Secreted
to mitochondrion
to plasma
membrane
to lysosome
to cytoplasm
to ECM
to clathrin-
coated pits
