# Supplementary material for: Identifying Therapeutic Targets for Amyotrophic Lateral Sclerosis Through Modeling of Multi-Omics Data
Source: Int J Mol Sci. 2025 Jul 23;26(15):7087. doi: 10.3390/ijms26157087 (PMC12346086; doi:10.3390/ijms26157087)
Supplement: Supplementary file 1 [file ijms-26-07087-s001.zip › 7 Blaudin de Th_et al. Supplementary Table 4.pdf]

**Supplementary Table 4:** Top enriched upstream regulators within the 753 input genes and the 326 candidate genes that have a significant Benjamini-Hochberg corrected P-value.		
	**753 input genes**	**326 candidate genes**
**Upstream regulators**	Beta-estradiol (2,37E-04)	Beta-estradiol (8,60E-11)
	GRN (2,37E-04)	IL4 (3,28E-09)
	LDB1 (6,73E-04)	APP (8,92E-08)
	LMO2 (6,73E-04)	GRN (1,83E-07)
	IL33 (1,09E-03)	TNF (1,83E-07)
