# Supplementary figures and images for: Identifying Therapeutic Targets for Amyotrophic Lateral Sclerosis Through Modeling of Multi-Omics Data
Source: Int J Mol Sci. 2025 Jul 23;26(15):7087. doi: 10.3390/ijms26157087 (PMC12346086; doi:10.3390/ijms26157087)

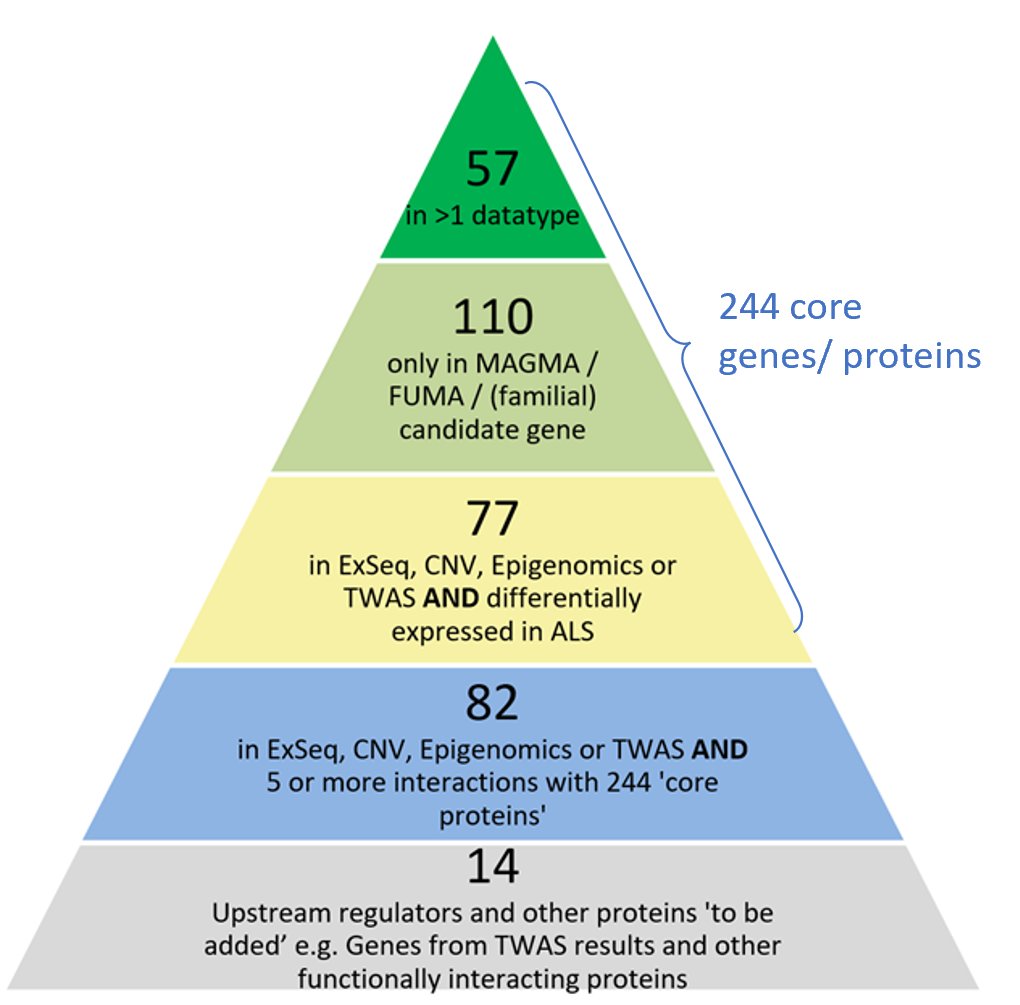

Supplement: Supplementary file 1 [file ijms-26-07087-s001.zip › 1 Blaudin de Th_et al. Supplementary Figure 1 - Figure 1a high quality.png]

## Slide 1
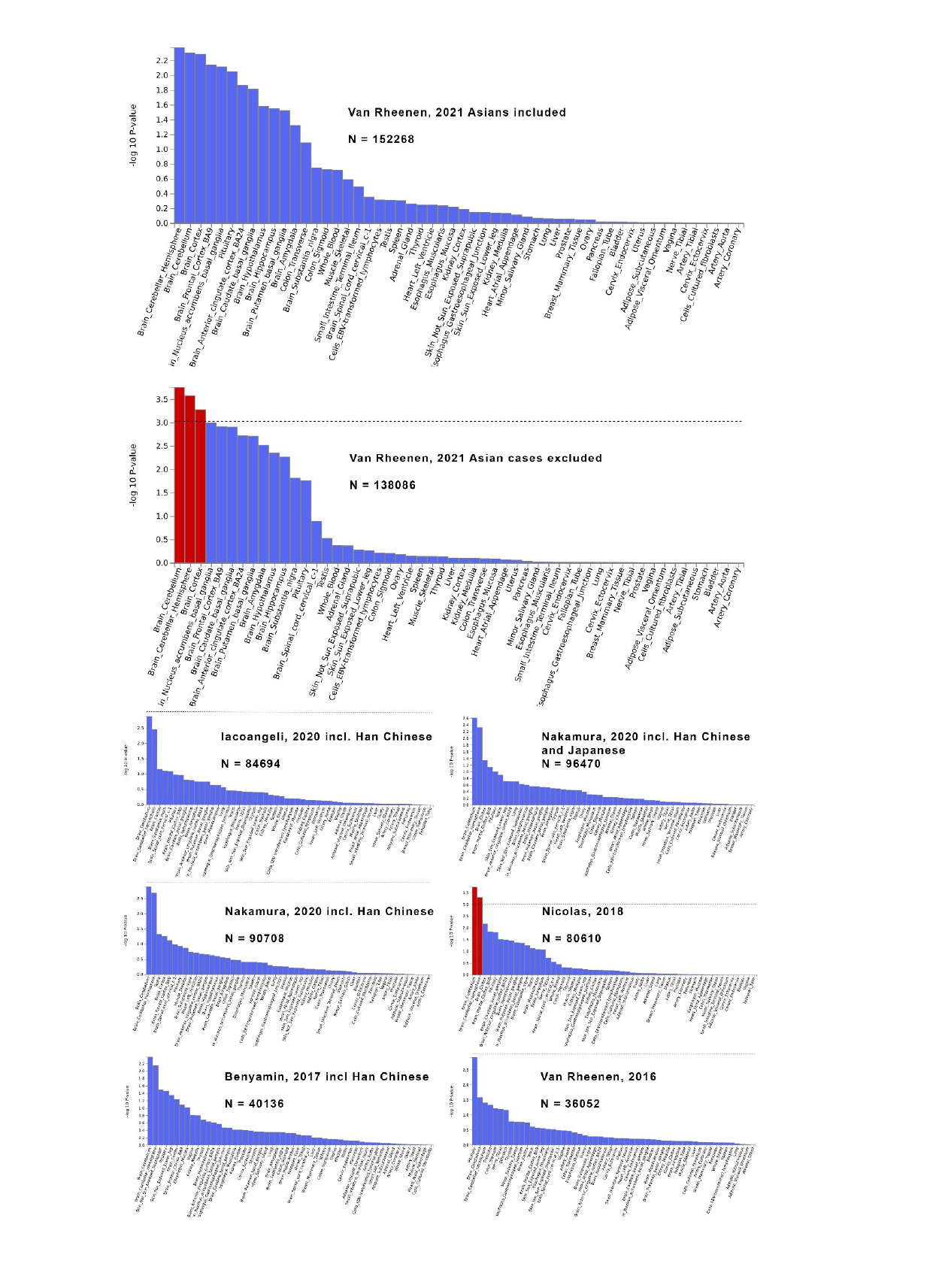

Supplement: Supplementary file 1 [file ijms-26-07087-s001.zip › 2 Blaudin de Th_et al. Supplementary Figure 2.pptx]

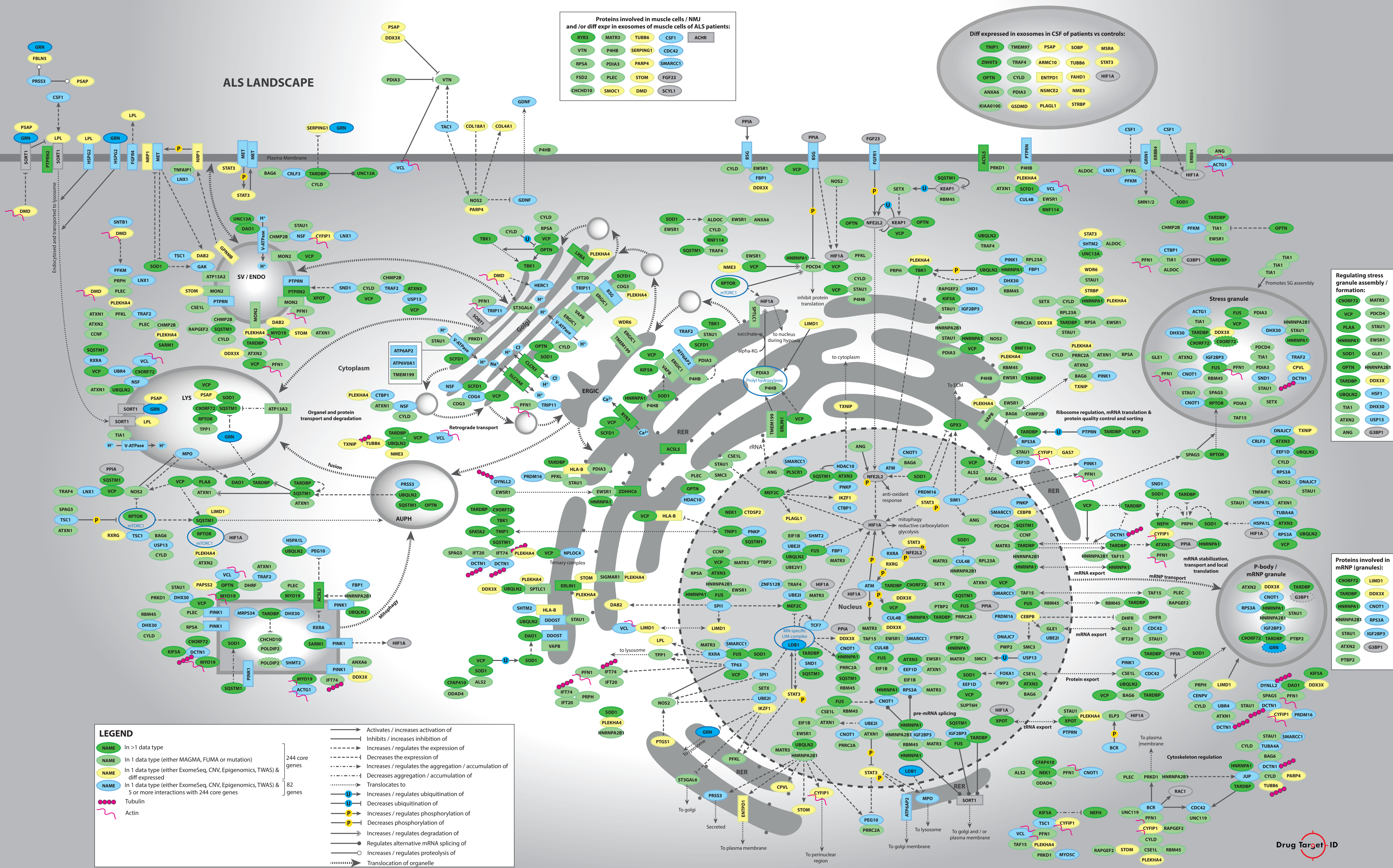

Supplement: Supplementary file 1 [file ijms-26-07087-s001.zip › 3 Blaudin de Th_et al. Supplementary Figure 3 - Figure 1b high quality.pdf]

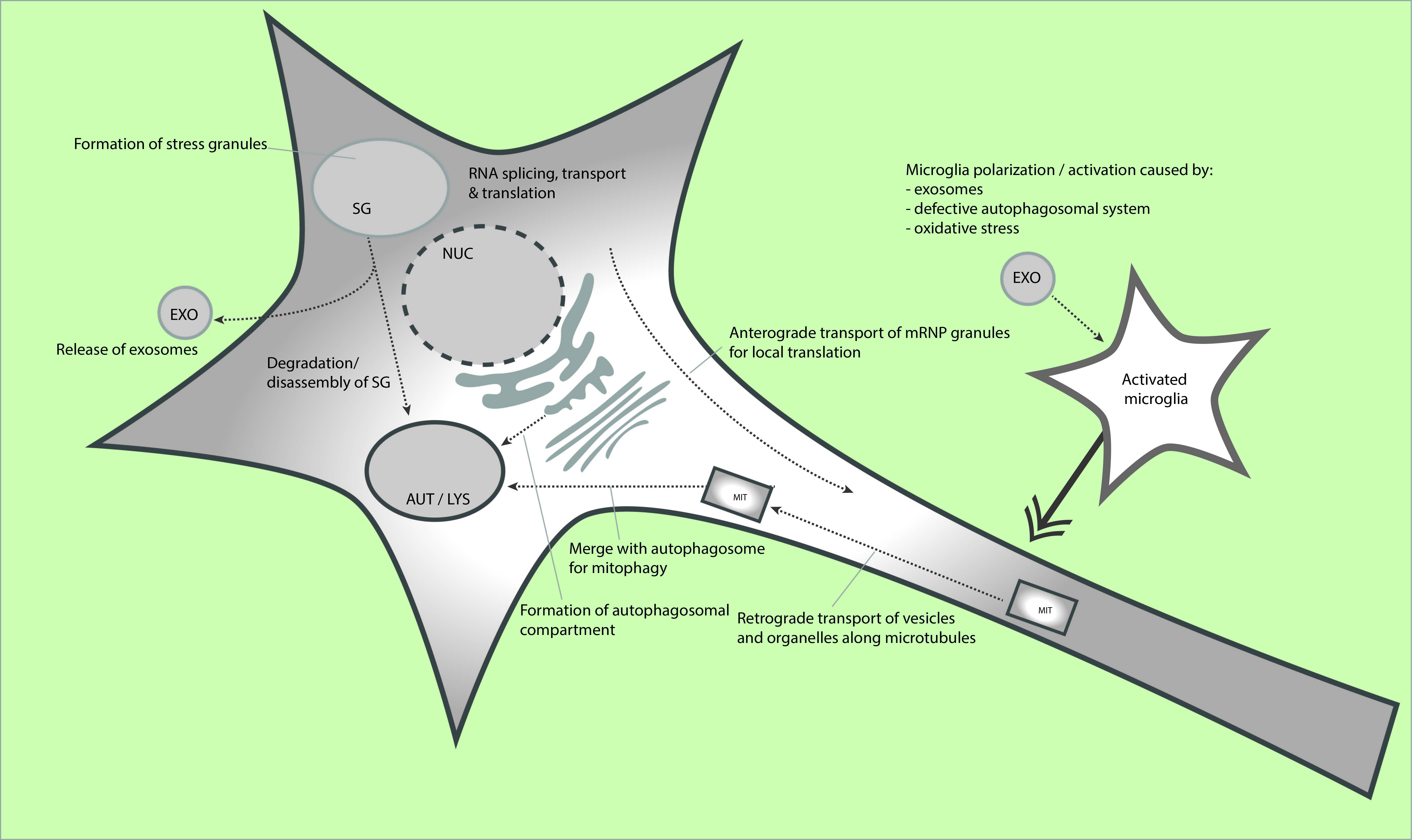

Supplement: Supplementary file 1 [file ijms-26-07087-s001.zip › 5 Blaudin de Th_et al. Supplementary Figure 5 - Figure 1c high quality.jpg]
